# High Prevalence of Persistent Parasitic Infections in Foreign-Born, HIV-Infected Persons in the United States

**DOI:** 10.1371/journal.pntd.0001034

**Published:** 2011-04-12

**Authors:** Natasha S. Hochberg, Ruth N. Moro, Anandi N. Sheth, Susan P. Montgomery, Frank Steurer, Isabel T. McAuliffe, Yun F. Wang, Wendy Armstrong, Hilda N. Rivera, Jeffrey L. Lennox, Carlos Franco-Paredes

**Affiliations:** 1 Department of Epidemiology, Boston University School of Public Health, Boston, Massachusetts, United States of America; 2 Division of Infectious Diseases, Emory University School of Medicine, Atlanta, Georgia, United States of America; 3 Rollins School of Public Health, Emory University, Atlanta, Georgia, United States of America; 4 Division of Parasitic Diseases and Malaria, Centers for Disease Control and Prevention, Atlanta, Georgia, United States of America; 5 Grady Memorial Hospital, Atlanta, Georgia, United States of America; 6 Department of Pathology and Laboratory Medicine, Emory University School of Medicine, Atlanta, Georgia, United States of America; 7 Hospital Infantil de Mexico Federico Gomez, Mexico City, Mexico; Case Western Reserve University School of Medicine, United States of America

## Abstract

**Background:**

Foreign-born, HIV-infected persons are at risk for sub-clinical parasitic infections acquired in their countries of origin. The long-term consequences of co-infections can be severe, yet few data exist on parasitic infection prevalence in this population.

**Methodology/Principal Findings:**

This cross-sectional study evaluated 128 foreign-born persons at one HIV clinic. We performed stool studies and serologic testing for strongyloidiasis, schistosomiasis, filarial infection, and Chagas disease based on the patient's country of birth. Eosinophilia and symptoms were examined as predictors of helminthic infection. Of the 128 participants, 86 (67%) were male, and the median age was 40 years; 70 were Mexican/Latin American, 40 African, and 18 from other countries or regions. *Strongyloides stercoralis* antibodies were detected in 33/128 (26%) individuals. Of the 52 persons from schistosomiasis-endemic countries, 15 (29%) had antibodies to schistosome antigens; 7 (47%) had antibodies to *S. haematobium*, 5 (33%) to *S. mansoni*, and 3 (20%) to both species. Stool ova and parasite studies detected helminths in 5/85 (6%) persons. None of the patients tested had evidence of Chagas disease (n = 77) or filarial infection (n = 52). Eosinophilia >400 cells/mm^3^ was associated with a positive schistosome antibody test (OR 4.5, 95% CI 1.1–19.0). The only symptom significantly associated with strongyloidiasis was weight loss (OR 3.1, 95% CI 1.4–7.2).

**Conclusions/Significance:**

Given the high prevalence of certain helminths and the potential lack of suggestive symptoms and signs, selected screening for strongyloidiasis and schistosomiasis or use of empiric antiparasitic therapy may be appropriate among foreign-born, HIV-infected patients. Identifying and treating helminth infections could prevent long-term complications.

## Author Summary

Undiagnosed and untreated parasitic infections can have severe consequences for human immunodeficiency virus (HIV)-infected persons. An estimated 2 billion people worldwide are infected with soil-transmitted helminths and schistosomiasis, yet there are few data on the prevalence in HIV-infected immigrants to more developed countries. This information could help clinicians determine what testing is needed and what signs or symptoms to expect. We performed serologic, stool, and urine testing for selected parasites in 128 foreign-born persons receiving care at an HIV clinic in Atlanta, Georgia. We found that 26% had serologic evidence of infection with *Strongyloides stercoralis* and 29% had serologic evidence of schistosomiasis. Because these were likely chronic processes, symptoms and signs were often absent; only weight loss was significantly associated with strongyloidiasis. High eosinophil counts were also associated with parasitic infection. This study suggests the need for targeted screening of foreign-born, HIV-infected persons for parasitic infections (mainly strongyloidiasis and schistosomiasis) or the use of empiric antiparasitic therapy, particularly among those with unexplained eosinophilia. Although there are established guidelines for screening of refugees, health care providers should consider the risk of these organisms in patients who have entered the United States through other pathways.

## Introduction

Human immunodeficiency virus (HIV) co-infection with parasitic diseases is an issue not only in parasite-endemic countries but also for persons who have migrated to more developed countries such as the United States. HIV surveillance systems in the United States do not routinely assess country of birth, so there are limited data on the number of foreign-born case patients [1]. In 2008, 19% of new HIV diagnoses were made among Hispanics (55% foreign-born), and data from Washington show foreign-born Blacks had HIV diagnosis rates 2.8 times higher than native-born Blacks [2], [3]. A study in sexually transmitted diseases clinics found equal HIV prevalence among native- and foreign-born persons [4]. Refugees, asylees, and immigrants are also at risk for strongyloidiasis, schistosomiasis, and other parasitic infections acquired in their countries of origin. An estimated 2 billion people worldwide are infected with soil-transmitted helminths and schistosomiasis; pathogenic intestinal parasite infections are commonly found in immigrants to developed countries including up to 98% of Ethiopian immigrants to Israel [5]–[7]. In the United States, an estimated 300,000 persons are infected with *Trypanosoma cruzi* [8].

Certain parasitic infections (e.g., *Cryptosporidium parvum*, *Isospora belli*, and *S. stercoralis*) are more prevalent among HIV-infected patients particularly those with lower CD4+ cell counts [9]–[11]. Furthermore, many parasitic infections can persist for decades and result in significant morbidity and mortality. Long-term consequences include schistosomiasis-associated portal hypertension, cardiac and gastrointestinal abnormalities from Chagas disease, and disseminated strongyloidiasis in the setting of human T-cell lymphotropic virus (HTLV) co-infection [12]–[14]. In addition, HIV-infected patients can have more severe and protean sequelae such as immune reconstitution syndromes from schistosomiasis and strongyloidiasis, rare but potentially fatal strongyloides hyperinfection, and more rapid development of hepatic fibrosis from schistosomiasis [6], [15]–[18]. Chagas disease reactivation in HIV-infected persons can be lethal with manifestations including meningo-encephalitis and acute myocarditis [19]–[21].

Despite the potentially severe complications from parasite co-infection with HIV, there are few data on the prevalence of parasitic disease in foreign-born persons with HIV residing in the United States. Furthermore, although there are guidelines for screening new refugees, there are no standards for how or when to look for parasitic infections among foreign-born persons with HIV who may have been in the United States for extended periods of time [5], [22]. Evaluating HIV-infected persons is complicated by the lack of sensitivity of stool testing in schistosomiasis and the many etiologies for eosinophilia in HIV-infected persons (including dermatologic disease and low CD4+ cell counts) in addition to those seen among HIV-uninfected persons (e.g. allergies, connective tissue disease) [23]–[25]. The first objective of the study was to determine the prevalence of selected parasitic infections among HIV-infected persons who live in the United States and were born in developing countries. Because of the frequency of comorbid illnesses resulting in abdominal symptoms or eosinophilia in HIV-infected persons, our second objective was to evaluate whether specific symptoms, signs or laboratory parameters are useful in predicting parasitic infection.

## Methods

### Ethics statement

Written informed consent was obtained from each participant; the study was approved by the Institutional Review Boards of Emory University and the Centers for Disease Control and Prevention (CDC). Each participant received $10 to compensate them for their time and the blood draw. Treatment for parasitic infections was not part of the study. Health care providers were informed of positive results and, when necessary, arranged treatment in conjunction with a tropical medicine expert.

### Study design

Between July–September 2009, we enrolled patients in a cross-sectional study at the Ponce de Leon Center in Atlanta, Georgia. This program provides care for ∼5,000 patients, most with nadir CD4+ cell counts less than 200 cells/mm^3^. On the day of their clinic visit, all non-pregnant, HIV-infected patients greater than 17 years of age who were born in a developing country were invited to participate. We questioned participants on demographics, risk factors for parasitic infection, and symptoms within the prior year. Medical records were reviewed for previous parasitic infection diagnoses, use of combination antiretroviral therapy (cART), physical examination findings from the last three clinic visits, and laboratory testing (including the three most recent complete blood counts [CBC] and a urinalysis for persons from schistosomiasis-endemic countries). Eosinophilia was defined as >400 eosinophils/mm^3^ on any of the CBCs; the highest absolute eosinophil count was used for analysis. Hematuria was defined as >2 red blood cells per high power field. The most recent CD4+ cell count and HIV viral load (VL) results were used for analysis.

### Laboratory testing

Serologic testing for parasitic diseases was performed at CDC in the Division of Parasitic Diseases and Malaria. All participants were tested for infection with *S. stercoralis* by enzyme immunoassay using soluble antigens from third-stage larvae [26]. Among persons with positive results on stool specimens, the test sensitivity is ∼95%, but the specificity has not been precisely determined for persons in non-endemic areas [13], [26]. For those with strongyloidiasis for whom serum was available, testing for HTLV was done at Quest Diagnostics (Tucker, GA). Persons from Mexico, Central America, and South America were tested for Chagas disease using an indirect fluorescent antibody (IFA) and a recombinant antigen enzyme linked immunosorbent assay (ELISA; Chagatest recombinante; Wiener, Argentina); polymerase chain reaction (PCR) testing and confirmatory trypomastigote excreted-secreted antigen-blot (TESA-blot) were performed for those with positive serological test results. For persons from schistosomiasis-endemic countries, Falcon assay screening test ELISA (FAST-ELISA) using *S. mansoni* adult microsomal antigen was done with subsequent *Schistosoma* enzyme-linked immunoelectrotransfer blot (immunoblot) species identification using *S. mansoni* and *S. haematobium* adult worm microsomal antigens. These assays have >95% sensitivity and nearly 100% specificity for both species [27], [28]. Urine was examined microscopically at CDC for ova and parasites and was tested for *Schistosoma* circulating cathodic antigen (CCA) (Rapid Medical Diagnostics, South Africa). Filarial IgG1 and IgG4 assays were performed for patients from endemic areas, and those with a history of seizures were tested for neurocysticercosis using *T. solium* immunoblot. Three stool samples per patient were examined for ova and parasites by concentrated wet mount and trichrome staining (Protocol Parasitology Systems, Thermo Scientific, Virginia) at the Grady Health Systems laboratory.

### Statistical analysis

The sample size was calculated based on the number of foreign-born persons who would come for a clinic visit during the study dates and agree to participate in the study. Data were entered into Excel 97-2003, and analysis was performed using SAS software, version 9.1 (SAS Institute). We used Fisher's exact test and Wilcoxon rank-sum test to compare the characteristics of persons with and without strongyloidiasis and schistosomiasis. Logistic regression modeling was used to evaluate predictors of strongyloidiasis, schistosomiasis, and eosinophilia. All clinically relevant and statistically significant (p≤0.05) variables in univariable analysis were initially included in the model and eliminated using a step-down model-building approach.

## Results

### Patient characteristics

Of the 128 persons in the study (80% participation rate among eligible persons invited to join the study), the median age was 40 years (range 19–64 years) and 86/128 (67%) were male (Table 1). Participants came from 41 birth countries: 48 (38%) were from Mexico; 40 (31%) from 20 African countries (including 8 from Ethiopia and 4 each from Ivory Coast, Kenya, and Nigeria); and 22 (17%) from 9 Central and South American countries. The median duration of time in the United States was 10.8 years (range 11 months–49 years); 19 (15%) persons were more recent arrivals (≤5 years in the country).

**Table 1:** Demographic and clinical characteristics of foreign-born, HIV-infected persons with and without parasite infections, 2009.

	Total (n = 128)	Infected with any parasite (n = 49)	Uninfected with any parasite (n = 79)
Age in years, median (range)	40 (19–64)	40 (21–62)	40 (19–64)
Male gender, N (%)	86 (67)	35 (71)	51 (65)
Regions/Countries of Origin, N (%)			
--Mexico	48 (38)	15 (31)	33 (42)
--Africa	40 (31)	21 (43)	19 (24)
---- Western Africa	18 (14)	9 (18)	9 (11)
---- Eastern Africa	14 (11)	8 (16)	6 (8)
---- Southern Africa	5 (4)	2 (4)	3 (4)
---- Central Africa	3 (2)	2 (4)	1 (1)
--Central America	16 (13)	7 (14)	9 (11)
--Caribbean	11 (9)	2 (4)	9 (11)
--Asia and Middle East	7 (6)	3 (6)	4 (5)
--South America	6 (5)	1 (2)	5 (6)
Years living in United States, median (range)	10.8 (0.9–48.7)	9.6 (1.3–35.6)	11.1 (0.9–48.7)
Years since HIV diagnosis, median (range)	5 (<1–21)	5 (<1–15)	5 (<1–21)
Years on cART, median (range)	4 (<1–18)	4 (<1–14)	4 (<1–18)
On cART at time of the study, N (%)^a^	112 (89)	43 (88)	69 (90)
CD4+ count (cells/mm^3^), median (range)	307 (0–1757)	194 (4–1159)	308 (0–1757)
CD4+ nadir (cells/mm^3^), median (range)	67 (0–982)^b^	79 (2–982)	64 (0–635)
Undetectable HIV VL, N (%)Detectable HIV VL (copies/ml), median (range)Eosinophil count (cells/mm^3^), median (range)	95 (74)31,245(120–564,000)196 (31–2304)	33 (67)18,800(120–529,000)264 (0–2304)	62 (78)41,000(230–564,000)176 (34–924)
Eosinophilia >400 cells/mm^3^, n (%)	25 (20)	15 (31)	10 (13)

HIV, human immunodeficiency virus; cART, combination antiretroviral therapy; VL, viral load.

a Data are missing for 2 persons.

b Although CD4+ cell count nadirs of <200 cells/mm^3^ or an AIDS diagnosis are generally required for entry into the clinic, we did not have all prior laboratory test results; therefore, some reported nadirs are >200 cells/mm^3^.

Although date of HIV infection was not known, HIV was diagnosed a median of 5 years before the study began (range 0–21years). The median CD4+ cell count was 307 cells/mm^3^ (range 0–1757), and 40/128 (31%) had CD4+ cell counts less than 200 cells/mm^3^. The median CD4+ cell count nadir was 67 cells/mm^3^ (range 0–982) (Table 1). At study enrollment, 113/128 (88%) were receiving cART; the median duration of time on cART was 4 years (range <1–18). The HIV VL was undetectable in 95/128 (74%) persons, and the median detectable VL was 31,245 copies/ml (range 120–564,000).

### Parasitic disease testing and clinical characteristics of infection

Serologic evidence of *S. stercoralis* infection was detected in 33 (26%) persons; none had larvae detected by stool examination. These persons were born in Mexico (n = 12), Honduras (n = 4), Ethiopia (n = 3), El Salvador (n = 2), Zambia (n = 2), and one each in Argentina, Congo, Cuba, Grenada, Guatemala, India, Kenya, Niger, Tanzania, and Vietnam. Strongyloidiasis-infected persons had been in the United States a median of 10.2 years; 4 (12%) were more recent arrivals. The most common symptoms and signs seen in persons with strongyloidiasis included weight loss (53%), diarrhea (48%), fatigue (42%), and abdominal pain (36%) (Table 2). Only weight loss was significantly associated with infection (OR = 3.1; 95% CI: 1.4–7.2) when controlling for CD4+ cell count and VL. None of the 26 strongyloidiasis-infected individuals tested for HTLV had evidence of infection. Those with strongyloidiasis had lower CD4+ cell counts (median 239; range 4–821) than those without the parasite (median 315; range 0–1757), but the difference was not significant (p = 0.16). Infection was also not associated with age, duration of time in the United States, or years since HIV diagnosis. Recalled history of walking barefoot and employment as a farmer in the country of birth were not statistically significant risk factors.

**Table 2:** Signs, symptoms, and laboratory findings of foreign-born, HIV-infected persons with and without parasite infection.

	Strongyloidiasis(n = 33)^a^	Schistosomiasis(n = 15)^a^	Persons without detectable parasite infection (n = 79)^b^
**Signs and Symptoms, n (%)**			
Weight loss	17 (53)	4 (27)	42 (41)
Diarrhea	16 (48)	5 (33)	26 (33)
Fatigue	14 (42)	6 (40)	29 (37)
Abdominal pain	12 (36)	4 (27)	15 (19)
Nausea or vomiting	5 (15)	4 (27)	20 (25)
Hematochezia	3 (9)	3 (20)	8 (10)
Abdominal tenderness on exam	3 (9)	0	5 (6)
Abdominal distension on exam	1 (3)	0	0
Hepatomegaly	1 (3)	0	1 (1)
Polyuria	NA	7 (47)	26 (33)
Hematuria	NA	1 (7)	2 (3)
Dysuria	NA	1 (7)	5 (6)
**Laboratory testing**			
CD4+ cell count (cells/mm^3^), median (range)	239 (4–821)	334 (4–596)	308 (0–1757)
CD4+ cell count nadir (cells/mm^3^), median (range)	68 (2–481)	54 (2–254)	64 (0–635)
Eosinophil count (cells/mm^3^), median (range)	256 (0–2,304)	320 (55–704)	176 (34–924)
Eosinophilia >400 cells/mm^3^, n (%)	8 (24)	7 (47)	10 (13)
Hematuria (documented)^c^	ND	4 (27)	ND

HIV, human immunodeficiency virus; NA, Not applicable; ND, Not done.

a 3 persons had schistosomiasis and strongyloidiasis and are included in both columns.

b Column totals do not add to 128, as persons with positive stool ova/parasite tests are not included in any column.

c Urinalysis results were only recorded for persons from schistosomiasis-endemic countries.

Among persons from endemic countries, schistosomiasis antibody testing was positive in 15/52 (29%); 7 (47%) had antibodies specific for *S. haematobium* antigens, 5 (33%) were reactive to *S. mansoni* antigens, and 3 (20%) had antibody reactivity to both species. Schistosomiasis had been previously diagnosed in 1/15 persons with *Schistosoma* antibodies (49 years earlier in Senegal); the patient was unsure whether he had been treated. Schistosome-reactive antibody positive persons had been in the United States a median of 6.8 years (including 6 more recent arrivals) and primarily came from Ethiopia (n = 3) and Ivory Coast (n = 2); the remainder came from other sub-Saharan African countries (Cameroon, Congo, Democratic Republic of the Congo, Liberia, Mali, Nigeria, Senegal, and Zambia) and one from Iran. Polyuria (47%), fatigue (40%), diarrhea (33%), weight loss (27%), and abdominal pain (27%) were the most common signs and symptoms in persons with *Schistosoma* antibodies, but none were significantly associated with a positive test for schistosome-reactive antibodies. Although 4/11 (36%) for whom prior urinalyses were available had documented hematuria, none had ova detected on urine microscopic examination; 3/13 (two with *S. mansoni* and one with *S. haematobium*) had detectable urine CCA. Positive schistosome serology was not associated with age, duration of residence in the United States, CD4+ cell count, or amount of time since HIV diagnosis. Recalled history of swimming or wading in water in the country of origin was not a significant risk factor.

No participant had evidence of filarial infection; 11/52 (21%) had equivocal or positive IgG1 testing (suggestive of exposure). Only 1/11 (9%) was also equivocally IgG4-positive (suggestive of possible infection), but the low IgG1 and equivocal IgG4 titers made true infection unlikely. Among 77 persons tested for Chagas disease (60 [78%] of whom reported living in thatch or adobe houses), 3 (4%) had positive ELISA results (range .414–.721; cutoff 0.3) but negative results on IFA, PCR, and TESA blots. The remaining 74 all tested negative by ELISA and IFA.

Stool samples were received from 85/128 (66%) participants; 5/85 (6%) had infection or co-infection with one of several pathogenic or potentially pathogenic intestinal parasites including four with *Entamoeba histolytica* and/or *E. dispar*, and one each with *Blastocystis hominis* and *Hymenolepis nana* [29]. Overall, 49/128 (38%) had evidence of parasitic infection by either serologic or stool tests. Positive testing for any parasitic infection was significantly associated with having resided in Africa (OR = 2.4; 95% CI: 1.1–5.1).

### Eosinophilia

The median eosinophil count was 196 cells/mm^3^ (range 0–2304), and 25/126 (20%) had eosinophilia (>400 eosinophils/mm^3^). Eosinophil counts were significantly higher among those with schistosome-specific antibodies than those without (median 320 v.142; p = .04) and among those with any one parasite infection compared to those without (264 v. 176; p = .02). Higher eosinophil counts were not significantly associated with strongyloidiasis, CD4+ cell count ≤200/mm^3^, or having a detectable HIV VL. Absolute eosinophilia (>400 cells/mm^3^) was significantly associated with having evidence of any parasite infection (p = .01) but not with a positive serology for schistosomiasis (p = .07) or comorbidities often associated with eosinophilia (eosinophilic folliculitis [n = 2], neoplasms [n = 7], asthma [n = 5], and connective tissue disease [n = 2]) or potentially associated with eosinophilia (seasonal allergies [n = 24]) [30]. In logistic regression modeling controlling for seasonal allergies, eosinophilia was associated with both schistosomiasis (OR = 4.5; 95% CI: 1.1–19.0) and low CD4+ cell counts (OR = 4.2; 95% CI: 1.0–17), although having a detectable HIV VL was not significantly associated. In a separate model, eosinophilia was associated with having any positive parasitic infection or serologic test when controlling for CD4+ cell count and seasonal allergies (OR = 3.0; 95% CI: 1.2–7.4).

## Discussion

Data have been lacking on the prevalence and indicators of subclinical parasitic infection in HIV-infected, foreign-born persons. To our knowledge, this is the first systematic study to use serologic, stool and urine testing to examine this population in the United States. We found that more than one-quarter of participants from endemic countries had antibodies to strongyloidiasis or schistosomiasis. Clinical signs and symptoms may not be useful predictors of chronic parasitic infection, but eosinophilia in foreign-born, HIV-infected persons should not be ignored, as it can signify schistosomiasis or another parasitic infection.

The high prevalence of antibody reactivity to parasitic infections found in our study of HIV-infected, foreign-born persons is similar to seroprevalences reported in other studies of migrant populations (HIV-infected and uninfected) [31]–[34]. We confirmed earlier findings that Africans may be at higher risk; Sarner et al. found that >50% of persons migrating from Africa have at least one positive parasite test, and others reported positive *Schistosoma* antibodies in 11–17% of HIV-infected African immigrants residing in the United Kingdom [31], . Sudanese and Somali Bantu refugees have particularly high rates of infection; *Schistosoma* antibodies have been found in 44% and 73% respectively (although HIV testing was either not performed or reported) [13]. The lack of *Trypanosoma cruzi* infections in our study is similar to findings from other reports describing Latin American immigrants [35]. Explanations include that we enrolled only 8 participants from countries with high seroprevalence (e.g. Bolivia, Argentina, Paraguay, El Salvador, and Honduras), we did not assess whether persons were from endemic areas within their countries of origin, and we included few older participants who are more likely to be seropositive [12], [36], [37]. The paucity of ova and parasites detected in the stool may result from the duration of time between likely exposure in the birth country and screening or from less intense infection [31].

Given the high prevalence of certain parasite infections in our study, our finding that eosinophilia was associated with positive schistosomiasis serology and any positive parasite test may be useful for HIV practitioners who must address the many possible causes of eosinophilia in their patients. This result confirms studies of African HIV-infected individuals in whom eosinophilia was associated with antibodies to both strongyloidiasis and schistosomiasis, but this has not been a consistent finding in other studies of HIV-infected persons [31], [34], [38]. Country of origin is clearly important when addressing causes of eosinophilia. Although high eosinophil counts have been associated with skin disease and advanced HIV raising the suggestion that parasite evaluation is not warranted, these investigators did not consider the patient's nationality [23], [24]. Furthermore, the relationship between eosinophilia and parasite infection status in our study may be less subject to misclassification bias, since more sensitive serologic testing was used in conjunction with stool studies that have poor sensitivity especially in HIV-infected persons with schistosomiasis [25].

In our study, clinical signs and symptoms were not reliable indicators of parasite co-infection. Other than weight loss in persons with strongyloidiasis, we found that no symptoms were significantly associated with parasite infection. The lack of associated abdominal pain has also been reported among HIV-uninfected refugees and may be due to the chronicity of parasite infection; associations with other signs and symptoms may not have been apparent because of the high prevalence of signs and symptoms due to other etiologies among HIV-infected persons without parasitic infections [13], [22].

Identifying and treating parasite co-infection in HIV-infected patients should be an important consideration for clinicians in developing and developed countries alike. Not only can HIV infection result in potentially severe complications of parasitic disease, but co-infection can affect HIV transmission and the disease course. *Schistosoma*-infected individuals may have increased HIV transmission rates because of elevated cytokine and HIV-infected white blood cell levels in the semen of infected men and ulcerative lesions in women with female genital schistosomiasis; HIV vertical transmission is also more likely in helminth-infected mothers [39]–[42]. In addition, co-infection may affect the course of HIV disease. Although some studies present evidence to the contrary, there are reports that treatment of helminth infections decreases HIV VL which could affect HIV transmission and time to AIDS-defining event or death [39], [43]–[47].

Although our study was strengthened by the use of serologic and urinary testing with excellent reported sensitivity and specificity, it is notable that test characteristics are undefined for the same assays when applied in patients with concomitant HIV [26], [28]. Similarly, persons co-infected with HIV and schistosomiasis excrete fewer eggs, so urine microscopy may be less sensitive in this setting [39]. Schistosomiasis serological assays are also limited by not allowing for differentiation between recent and old (potentially treated or cleared) infections. It is therefore possible that we overestimated infection rates, but this is unlikely because only one participant reported possible treatment for schistosomiasis (and this was almost 50 years prior). The remaining 14 (none of whom recalled a diagnosis of or treatment for schistosomiasis) may have had persistent or cleared infections. Cysticercosis serologic results may have underestimated larval *T. solium* infection rates, as we only tested persons with seizures, the most common manifestation of neurocysticercosis; we can not make generalizations regarding the overall prevalence of cysticercosis in the population studied. Lastly, it is possible but unlikely that strongyloidiasis was acquired after arrival in the country; to our knowledge, the last cases of domestic infection were reported in 1981, but sexual transmission has been reported [48], [49].

Our study had other limitations. As is the case for retrospective studies, our reliance on medical records for physical examination data might have decreased our ability to detect associations between infection and subtle clinical signs. Our sample size was small, so we had low statistical power to detect associations between infection and symptoms, signs and laboratory findings; the sample size is comparable to previous studies and was necessitated by the extensive serologic testing performed [23], [24], [26]. Due to the descriptive nature of the study, we did not control for multiple comparisons. Finally, with respect to the generalizability of our findings, almost all persons receiving care at the study site had nadir CD4+ cell counts less than 200 cells/mm^3^, and we had a large proportion of persons from Mexico; our results may therefore not be fully applicable to the HIV-infected population with higher CD4+ cell counts or from other countries. Additional information about patients who chose not to participate would also have helped us assess generalizability.

We found that HIV-infected, foreign-born persons are at high-risk for sub-patent, chronic parasitic infections, particularly strongyloidiasis and schistosomiasis, and classic symptoms and signs may be lacking. Based on our study findings, we suggest the implementation of targeted screening for foreign-born, HIV-infected persons. Because of the risk of undiagnosed and untreated disease, we recommend evaluation for or empiric guideline-directed treatment of strongyloidiasis and schistosomiasis for all persons from endemic areas, particularly those with eosinophilia [50]. Some studies suggest that empiric therapy may be more cost-effective than testing [51], [52]. Screening for Chagas disease has been recommended in patients with potential exposure history such as immigrants from endemic parts of Mexico, Central, and South America; other information on testing options and country-specific parasitic disease risk is readily available [12], [53]–[56]. Although there are established guidelines for screening refugees for parasitic infections, health care providers should consider the risk of these infections in persons who have entered the United States through other pathways, particularly those with HIV infection [13], [22].
